# Fifteen Years of Non-Hodgkin Lymphoma in an Indonesian National Referral Hospital: Epidemiologic Trends and Diagnostic Challenges

**DOI:** 10.1200/GO-24-00346

**Published:** 2024-11-07

**Authors:** Agnes Stephanie Harahap, Maria Francisca Ham, Andree Kurniawan, Stefanny Charles, Felix Wijovi, Lugyanti Sukrisman

**Affiliations:** ^1^ Anatomical Pathology Department, Faculty of Medicine, Universitas Indonesia/Dr. Cipto Mangunkusumo National Central General Hospital, Jakarta, Indonesia; ^2^ Human Cancer Research Center-Indonesia Medical Education and Research Institute, Faculty of Medicine, Universitas Indonesia, Jakarta, Indonesia; ^3^ Subspecialist Program Study, Hematology and Medical Oncology, Internal Medicine Department, Faculty of Medicine, Universitas Indonesia/Dr. Cipto Mangunkusumo National Central General Hospital, Jakarta, Indonesia; ^4^ Hematology and Medical Oncology, Internal Medicine Department, Faculty of Medicine, Universitas Indonesia/Dr. Cipto Mangunkusumo National Central General Hospital, Jakarta, Indonesia

## Abstract

**PURPOSE:**

The global burden of lymphoma is substantial because of the increase in its incidence in recent decades. However, disease characteristics vary across different geographical locations. Numerous immunohistochemistry markers and molecular studies are essential to determine lymphoma diagnosis and prognosis. This poses significant challenges in developing countries with limited health care resources. This large-scale study assesses the frequency of non-Hodgkin lymphoma (NHL) in Indonesia over the past 15 years, analyses its clinicopathologic features, and predicts future trends.

**METHODS:**

This retrospective study collected lymphoma patients diagnosed at the Department of Anatomical Pathology Dr. Cipto Mangunkusumo National Central General Hospital, Indonesia, from 2009 until 2023. All lymphoma diagnoses were confirmed by using ancillary tools classified as an enhanced lymphoma panel according to a resource-stratified guideline. We analyzed the clinicopathologic features of each NHL type and further applied the Autoregressive Integrated Moving Average model to predict future incidence trends.

**RESULTS:**

The study consisted of 7,368 NHL patients. Among these, B-cell lymphomas accounted for 90.6%, with diffuse large B-cell lymphoma being the most prevalent subtype (68.8%), followed by follicular lymphoma (8.8%) and marginal zone lymphoma (5.8%). Extranodal natural killer/T-cell lymphoma, nasal type, is the most common T-cell lymphoma found (26.3%). All types of lymphoma were found to be more common in males (57.7%). Extranodal involvement, particularly in the tonsil and upper respiratory tract, was frequently observed. Projection analysis indicates a steady increase in lymphoma patients in the future.

**CONCLUSION:**

This study highlights the distribution and burden of NHL in Indonesia over 15 years. The overall epidemiologic pattern of NHL in this study aligns with the results observed in other Asian countries. The rising incidence of lymphoma requires improved health care infrastructure and prevention strategies.

## Teaser

This study highlights the distribution and burden of NHL in Indonesia over 15 years and aligns with the results observed in other Asian countries. The rising incidence of lymphoma requires improved health care infrastructure and prevention strategies.

## INTRODUCTION

Lymphoma is a heterogeneous hematologic malignancy broadly classified into non-Hodgkin lymphoma (NHL) and Hodgkin lymphoma (HL). Because of the consistent increase in its incidences in most geographic regions, the global burden has become significant.^1,2^ In 2022, NHL ranked as the tenth most prevalent cancer worldwide.^3^ The global number of NHLs is expected to continue rising and is projected to reach 778,000 by the year 2040.^1^ The distribution and characteristics of lymphoma varied according to different regions and nations. Incidence rates were highest in Europe, Northern America, and East Asia countries.^3-6^ By contrast, low- to average-income countries, such as the countries from Africa and Southeast Asia, exhibited greater mortality rates.^3,6,7^ The distinct distribution of each lymphoma type has also been noted in various regions.^1^ In Southeast Asian countries, such as Thailand, the median age of patients with NHL is younger compared with Caucasians.^6^ Furthermore, the distribution of lymphoma subtypes in Asia is more varied than in North American and Western European countries, with higher incidences of mature extranodal natural killer (NK)/T-cell lymphoma, nasal type, and lower rates of follicular lymphoma (FL) and HL.^8^ Aside from variations in genetic and environmental exposure, a disparity in health care resources that causes suboptimal diagnosis and treatment of lymphoma may also contribute to this phenomenon.^3,9^

**CONTEXT**

**Key Objective**
How does this study provide new insights into the rising incidence and diagnostic challenges of non-Hodgkin lymphoma (NHL) in Indonesia, specifically in limited health care resources and how these constraints affect accurate diagnosis?
**Knowledge Generated**
This study investigates the patterns of incidence and difficulties in diagnosis associated with NHL in Indonesia over 15 years. B-cell lymphomas make up 90.6% of all patients with dominated diffuse large B-cell lymphoma subtypes. Natural killer/T-cell lymphoma, nasal type, is the predominant subtype of T-cell lymphoma. The findings show NHL more common in male adults age 50-59 years. The study uses the autoregressive integrated moving average model to forecast trend in NHL incidence, emphasizing the urgent need to improve health care infrastructure and develop comprehensive public health policies.
**Relevance**
The increasing prevalence of NHL emphasizes the need to improve health care infrastructure. Health authorities can use these data to allocate resources in a more effective way for improving access to diagnostic and specialized care.

To establish a definite diagnosis on the basis of the current classification, complex laboratory techniques ranging from immunohistochemistry (IHC) with numerous markers to molecular examinations are necessary to determine the lymphoma diagnosis and prognosis. Especially in low-income to middle-income developing nations, the diagnosis of lymphomas can be a particularly difficult task. In 2013, Tan et al^5^ published a resource-stratified guideline for lymphoma diagnosis and management. This approach was used to sufficiently unify the diagnostic and therapeutic strategies in several Asian countries with different levels of health care resources.

Indonesia, a diverse Southeast Asian nation of 270 million, has a growing aging population, increasing the incidence of chronic diseases such as cancer. The rising demand for health care services makes understanding the national burden of lymphoma critical for public health strategies. However, comprehensive data on lymphoma incidence, subtypes, and clinical features remain limited, particularly in large-scale data sets, making it essential to address this gap for effective disease management. This study aimed to describe a 15-year incidence of NHL at Dr. Cipto Mangunkusumo National Central General Hospital, its clinicopathologic characteristics, and further analyze future trends of lymphoma incidence.

## METHODS

### Ethics

The study received ethical approval from the Research Ethics Committee of the Faculty of Medicine, Universitas Indonesia—Dr. Cipto Mangunkusumo Hospital (UI-CMH) under protocol number KET-1317/UN2.F1/ETIK/PPM.00.02/2023, along with informed consent waiver permission number ND-2/UN2.F1/ETIK/PPM.00.02/2023.

### Study Design

A retrospective analysis was conducted on all NHL patients diagnosed between 2009 and 2023 from the Department of Anatomical Pathology, UI—CMH. Diagnosis was confirmed through hematoxylin and eosin staining, IHC, and Epstein-Barr virus-encoded RNA-chromogenic in situ hybridization (EBER-ISH) for selected patients. The characteristics of the antibodies are provided in the Data Supplement. Lymphomas were classified following the latest WHO guidelines.^10^ Lymphoma patients that could not be categorized into specific entities because of a lack of markers were classified as unclassifiable B-cell lymphoma and unclassifiable T-cell lymphoma.

Clinical data, including patient age, sex, disease onset, and tumor characteristics, were obtained from medical records. Tumor location was classified as nodal, extranodal, or both. Nodal sites were further divided into upper, lower, or both diaphragmatic regions. Extranodal involvement was classified by specific locations such as brain, head and neck, thorax, abdomen, genitalia (male and female), urinary system, bone, soft tissue, bone marrow, and skin.

### Statistical Analysis

Data were analyzed using Microsoft Excel 2023, Statistical Package for the Social Science 26.0, and Python. The autoregressive integrated moving average (ARIMA; p, d, q) model was used to predict lymphoma incidence and future trends, incorporating autoregression (p), moving average (q), and differencing (d) to stabilize time-series data. The annual percentage change (APC) was calculated on the basis of year-to-year patient differences.

## RESULTS

### Basic Characteristics and Tumor Types

**FIG 1:**
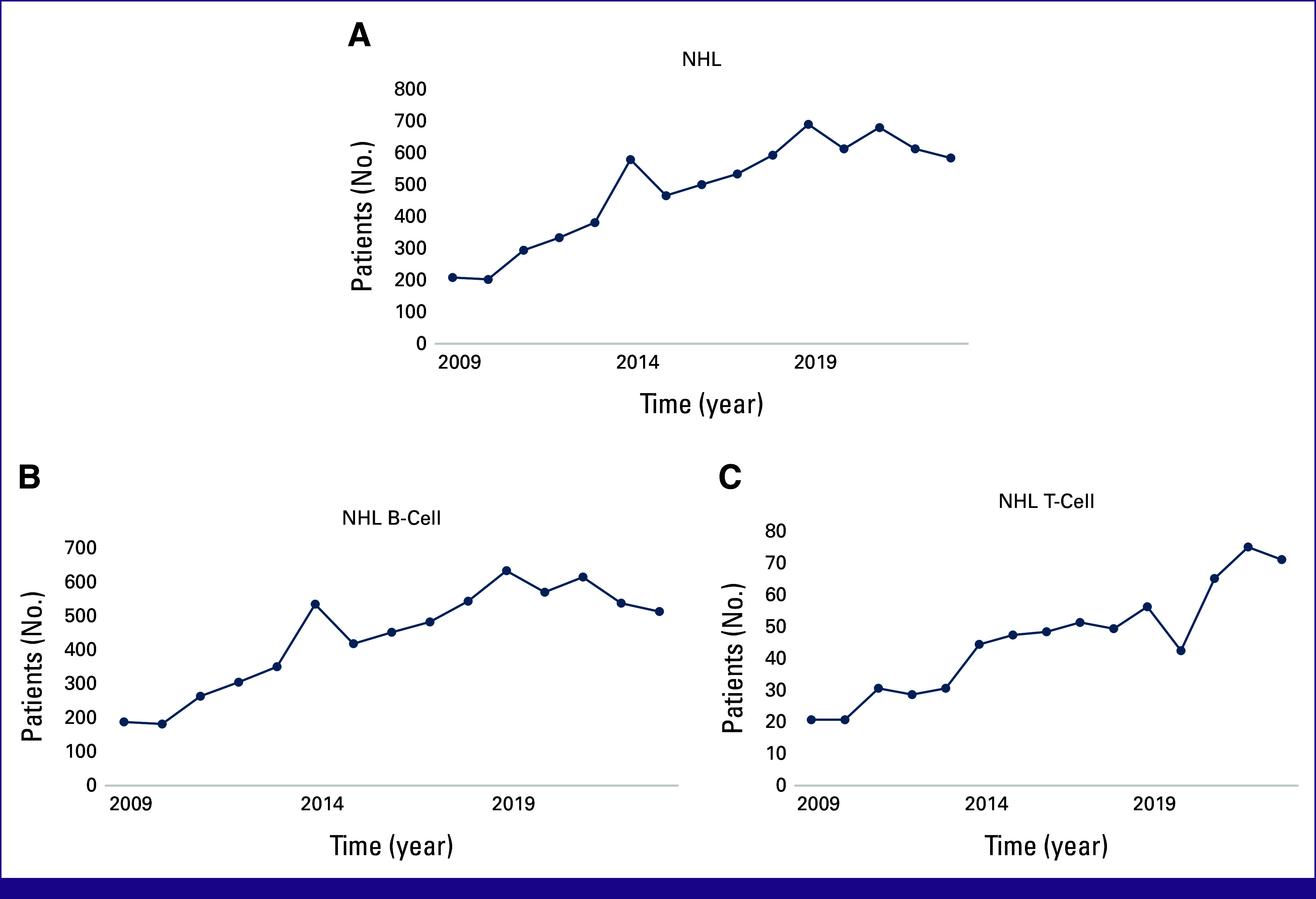
Annual distribution of total (A) NHL, (B) NHL B-cell, and (C) NHL T-cell. NHL, non-Hodgkin lymphoma.

During the 15-year study period, a total of 13,224 lymphoma patients were identified. Of these, 4,963 patients were excluded because of incomplete diagnostic panels or uncertain diagnosis. Furthermore, 893 patients of HL were eliminated, resulting in a final cohort of 7,368 patients included in the study analysis. Notably, 47.6% of the patients originated from our institution, while 52.5% were referred from other hospitals. The peak incidences of NHL patients were seen during 2014, 2019, and 2021 (Fig 1).

NHL constituted 89.2% of the overall patients with lymphoma. B-cell lymphoma accounted for 90.6%, while T-cell lymphoma accounted for 9.4%. Table 1 shows that there is a slight majority of males in the NHL (51.8%). The majority of B- and T-cell lymphomas were diagnosed in individuals age 50-59 and 40-49 years, respectively.

**TABLE 1:** Description of the Basic Characteristics of NHL Patients

Characteristic	NHL (N = 7,368)		
	B-Cell Lymphoma (n = 6,677), No. (%)		T-Cell Lymphoma (n = 691), No. (%)
Sex		Sex	
Male	3,822 (57.2)	Male	459 (66.4)
Female	2,855 (42.8)	Female	232 (33.6)
Age, years		Age, years	
<10	156 (2.3)	<10	39 (5.6)
10-19	146 (2.2)	10-19	76 (11)
20-29	340 (5.1)	20-29	75 (10.9)
30-39	689 (10.3)	30-39	110 (15.9)
40-49	1,338 (20)	40-49	129 (18.7)
50-59	1,742 (26.1)	50-59	122 (17.7)
60-69	1,466 (22.1)	60-69	83 (12)
70-79	657 (9.8)	70-79	50 (7.2)
>80	143 (2.1)	>80	7 (1)
Subtype	6,677 (100)	Subtype	691 (100)
Precursor/lymphoblastic	70 (1.1)	Precursor/lymphoblastic	122 (17.7)
Mature		Mature	
SLL/CLL	55 (0.8)	Extranodal NK/T, nasal type	182 (26.3)
MCL	253 (3.8)	NK/T, nodal	3 (0.43)
Follicular	584 (8.8)	Mycosis fungoides	45 (6.51)
MZL	390 (5.8)	Lymphomatoid papulosis	4 (0.58)
Lymphoplasmacytic	30 (0.5)	ALCL	159 (23.0)
PCFCL	8 (0.1)	ALK-positive	66 (41.5)
DLBCL	4,592 (68.8)	ALK-negative	64 (40.3)
GCB	1,023 (22.3)	NA	29 (18.2)
Non-GCB	1,972 (42.9)	AITL	44 (6.4)
NA	1,597 (34.8)	PTCL-NOS	60 (8.7)
THRLBCL	6 (0.1)	MEITL	5 (0.7)
Plasmablastic	40 (0.6)	UCT	67 (9.7)
Primary mediastinal large B-cell	101 (1.5)		
Gray zone	16 (0.2)		
Burkitt	214 (3.2)		
UCB	318 (4.8)		

Abbreviations: AITL, angioimmunoblastic T-cell lymphoma; ALCL, anaplastic large cell lymphoma; ALK, anaplastic lymphoma kinase; CLL, chronic lymphocytic lymphoma; DLBCL, diffuse large B-cell lymphoma; GCB, germinal center B-cell-like; MCL, mantle cell lymphoma; MEITL, monomorphic epitheliotropic intestinal T-cell lymphoma; MZL, marginal zone lymphoma; NA, not available; NHL, non-Hodgkin lymphoma; NK/T, natural killer/T-cell lymphoma; PCFCL, primary cutaneous follicle center lymphoma; PTCL-NOS, peripheral T-cell lymphoma, not otherwise specified; SLL, small lymphocytic lymphoma; THRLBCL, T-cell histiocyte-rich large B-cell lymphoma; UCB, unclassifiable B-cell lymphoma; UCT, unclassifiable T-cell lymphoma.

Table 1 presents the distribution of each type of NHL. B-cell NHL is predominantly represented by diffuse large B-cell lymphoma (DLBCL), followed by FL, marginal zone lymphoma (MZL), mantle cell lymphoma (MCL), and Burkitt lymphoma. The most prevalent subtypes of DLBCL, on the basis of the cell they originate from, are nongerminal center B-cell–like subtypes (non-GCB). Among the immature lymphoma, T-ALL was found more often than B-ALL. A total of 1,095 patients with DLBCL underwent CD30 IHC staining, in which 171 patients (15.5%) showed positive staining. Most FL tumors (319, 54.7%) are low-grade. However, among the transformed patients with DLBCL, FL was the most commonly found (90.3%).

The T-cell NHL subtype is dominated by extranodal NK/T-cell lymphoma, nasal type, followed by anaplastic large cell lymphoma (ALCL). Of a total of 130 patients of ALCL that underwent IHC staining for the anaplastic lymphoma kinase (ALK) marker, 66 patients (41.5%) exhibited positive staining.

### Age Distribution of Lymphoma

**FIG 2:**
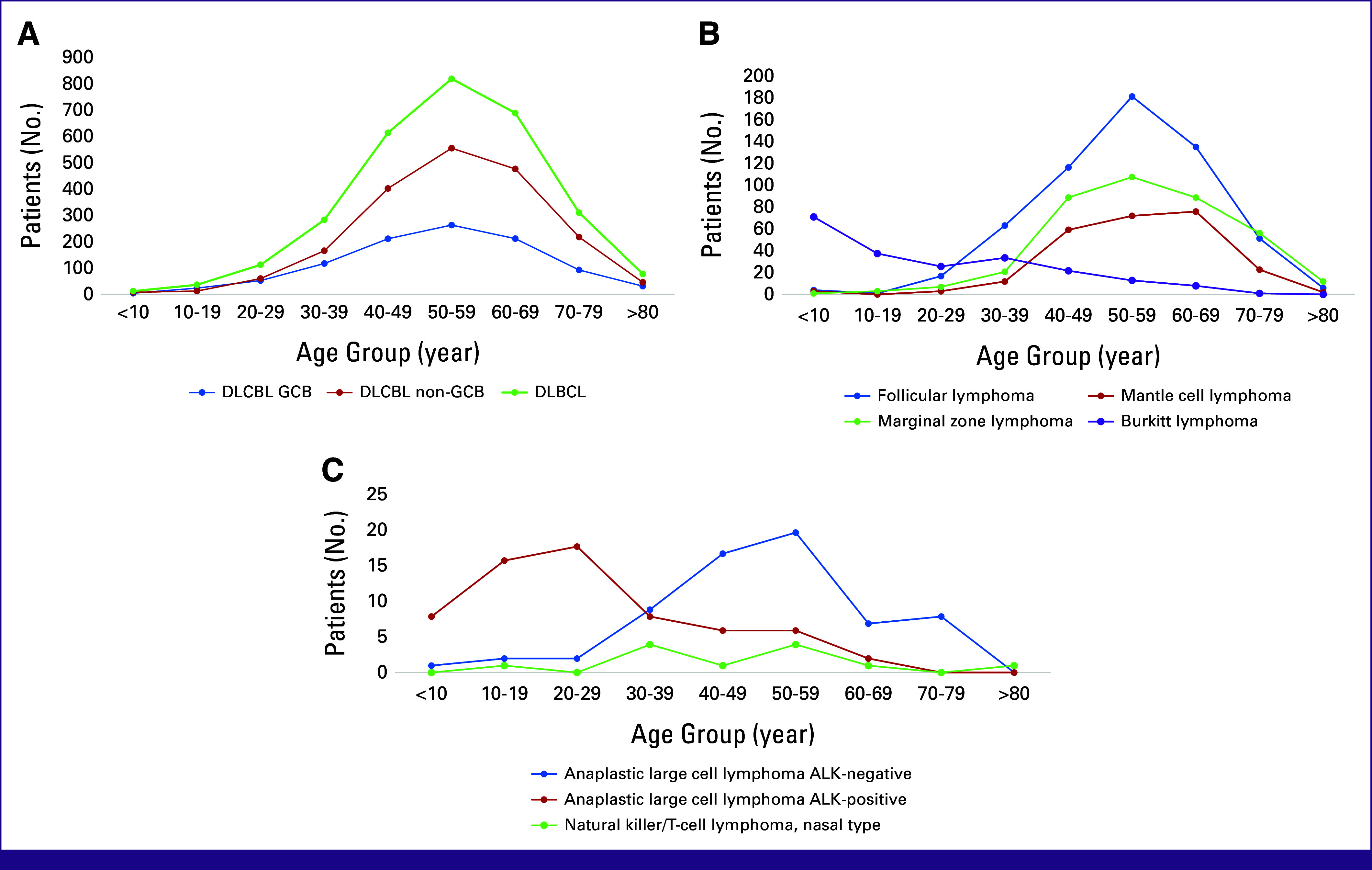
(A) Age distribution of DLBCL. Non-GCB and GCB subtypes of DLBCL showed no differences in age distribution. (B) Age distribution for the other type of B-cell NHL. (C) Age distribution for T-cell NHL. ALK, anaplastic lymphoma kinase; DLBCL, diffuse large B-cell lymphoma; GCB, germinal center B-cell-like; NHL, non-Hodgkin lymphoma.

As displayed in Figure 2, each lymphoma type showed a different age distribution. The peak incidences of DLBCL, FL, and MZL were in the 50-59 age group, whereas MCL was found in the 60-69 age group. Burkitt lymphoma showed two incidence peaks, which occurred in the <10 years and 30-39 age groups. ALCL-ALK–negative showed peak incidence in the 50-59 age group, whereas ALCL-ALK–positive had peak incidence in the 20-29 age group.

### Gender Distribution of Lymphoma

**FIG 3:**
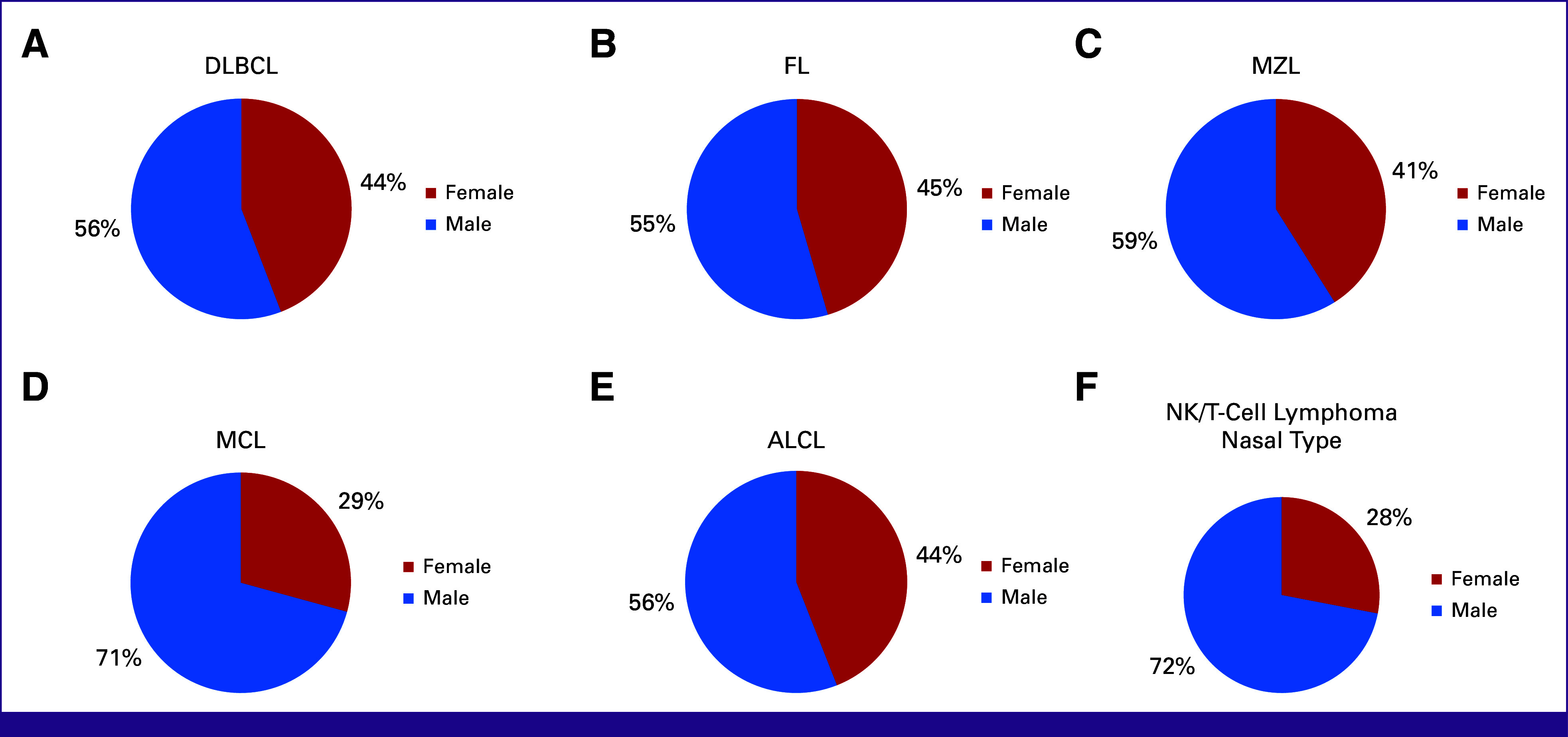
Gender distribution in (A) DLBCL, (B) FL, (C) MZL, (D) MCL, (E) ALCL, and (F) NK/T-cell lymphoma nasal type. ALCL, anaplastic large cell lymphoma; DLBCL, diffuse large B-cell lymphoma; FL, follicular lymphoma; NK, natural killer; MCL, mantle cell lymphoma; MZL, marginal zone lymphoma.

In this study, males were the predominant sex with 51.8% in all patients with NHL. As presented in Figure 3, DLBCL, FL, MZL, and ALCL have similar male-to-female ratios, which are 56:44, 55:45, 59:41, and 56:44, respectively. The male-to-female ratios for MCL and NK/T nasal type of lymphoma reached 71:29 and 72:28, respectively.

### Tumor Site Predilection

Among all lymphoma patients, a slight majority were located in extranodal sites (53.3%). Specifically, 51.2% of B-cell NHLs and 58.2% of T-cell NHLs were found in extranodal locations. Meanwhile, nodal predilection was found in 37.2% of B-cell NHL and 31.4% of T-cell NHL. Table 2 shows the top three distributions of tumor locations and predilection sites for different lymphoma types. Tonsil is the most common extranodal site affected, particularly in DLBCL, FL, and MCL.

**TABLE 2:** The Top Three Most Common Lymphoma Sites

Lymphoma Type	Extranodal (%)	Nodal (%)	Lymphoma Type	Extranodal (%)	Nodal (%)
DLBCL	65.0	35.0	MZL	85.3	14.7
	Tonsil (14.9)	Cervical (58.2)		Eyes (63.4)	Cervical (73.7)
	URT (12.7)	Inguinal (12.4)		Head and neck (5.4)	Submandibular (12.3)
	Colon (9.0)	Axillary (6.9)		Stomach (4.5)	Inguinal (7.0)
FL	33.5	66.5	EN NK/T-CL	97.7	2.3
	Tonsil (17.4)	Cervical (42.7)		Nose and sinus (66.9)	Cervical (75.0)
	Salivary glands (7.9)	Inguinal (25.0)		URT (14.5)	Inguinal (25.0)
	Colon (5.6)	Axillary (8.1)		Eyes (2.9)	55.9
MCL	48.8	51.2	ALCL	44.1	Cervical (43.0)
	Tonsil (22.9)	Cervical (48.2)		Skin (19.0)	Inguinal (26.6)
	URT (14.3)	Inguinal (13.6)		Upper extremities (15.9)	Axillary (10.1)
	Colon (11.4)	Axillary (7.3)		Lower extremities (7.9)	

Abbreviations: ALCL, anaplastic large cell lymphoma; DLBCL, diffuse large B-cell lymphoma; EN NK/T-CL, extranodal natural killer/T-cell lymphoma nasal type; FL, follicular lymphoma; MCL, mantle cell lymphoma; MZL, marginal zone lymphoma; URT, upper respiratory tract.

### Incidence of Lymphoma and Future Trend

**FIG 4:**
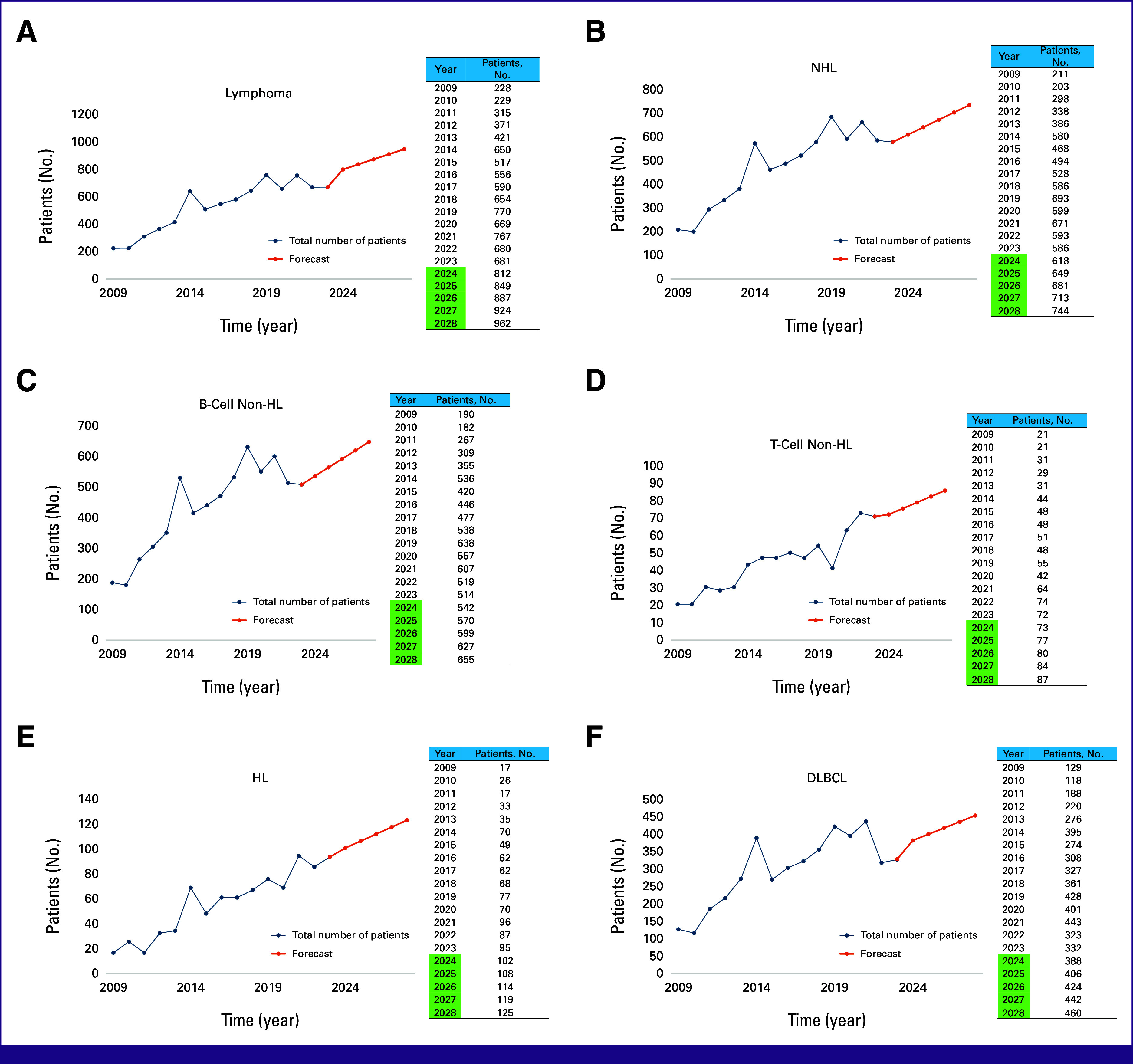
Future trend of (A) all patients with lymphoma, (B) NHL, (C) B-cell lymphoma, (D) T-cell lymphoma, (E) HL, and (F) DLBCL. DLBCL, diffuse large B-cell lymphoma; HL, Hodgkin lymphoma; NHL, non-Hodgkin lymphoma.

Figure 4 illustrates the projected incidence trend of lymphoma patients by using the ARIMA model (0,1,1) (1,1,0). The data suggest an increasing trend of patients with NHL and HL. The average APC of patients with lymphoma was 9.1%, with NHL at 8.6%, B-cell lymphoma at 8.5%, T-cell lymphoma at 10.4%, HL at 19.0%, and DLBCL at 8.9% from 2009 to 2023. The average APC of all lymphoma types is projected to increase 7.3% in the next 5 years. Specifically, patients with NHL are likely to rise by 4.9%, patients with B-cell lymphoma by 5.0%, patients with T-cell lymphoma by 3.9%, patients with HL by 5.7%, and patients with DLBCL by 6.9% on average each year over the next 5 years.

A t-test analysis of the incidence rates by sex, as indicated by the annual changes, was performed and showed no statistically significant difference between the two groups (*P* = .233).

## DISCUSSION

NHL is significantly more prevalent than HL, accounting for approximately 90% of patients with lymphoma worldwide.^11,12^ In this study, NHL comprised 89.2% of all patients with lymphoma. This finding aligns with previous studies from China and South Korea, where NHL accounted for 87%-95.9% of all patients with lymphoma.^13-15^ Hitherto, to our knowledge, this is the largest Indonesian epidemiologic study on lymphoma by sample size, exceeding a previous cohort documenting 761 patients with 93.9% classified as NHL.^16^ Contrastingly, HL is more common in Pakistan, Iran, and India, comprising 25.3%-30.6% of patients, while in our study, it accounted for only 10.8%.^17-19^ These variations may result from different sample sizes, ethnicities, risk factors (like Epstein-Barr virus), and geographical differences, warranting further investigation.

Lymphoma is a heterogeneous hematologic malignancy with over 70 subtypes ranging from indolent to aggressive neoplasms. Accurate subtype diagnosis typically requires IHC, EBER-ISH, molecular analysis, and genetic profiling. However, limited health care resources in developing countries hinder comprehensive diagnosis. In Asia, B-cell lymphoma diagnostic strategies are classified into four tiers on the basis of resource availability: basic, limited, enhanced, and maximum.^5^ The CMH offers an enhanced diagnostic panel, one of the few in Indonesia capable of performing adequate lymphoma examinations.^5^

Previous literature has shown that B-cell lymphoma comprises 85%-90% of adult NHL.^20,21^ This accords with our observation in which 90.6% of NHLs were classified as B-cell lymphoma. Consistent with previous studies,^22^ DLBCL remains the most frequent entity for mature B-cell, followed by FL, MZL, MCL, and Burkitt lymphoma. Gene expression profiling is considered the most reliable method for subtyping DLBCL into GCB and non-GCB subtypes, which is critical as the GCB subtype has a better prognosis. Using the Hans algorithm, we found a higher prevalence of non-GCB compared with GCB, aligning with previous reports that show non-GCB subtypes in 60%-72% of DLBCL patients.^23-25^ GCB subtypes are less common in Asia than in Western countries, possibly because of genetic polymorphisms or differences in diagnostic practices.^23,26^ This is particularly evident as the subtypes of 34.8% of patients with DLBCL in this study could not be classified because of incomplete result of CD10, BCL6, and MUM1 markers. CD30 expression was observed in 15.5% of patients with DLBCL in our study, consistent with the 10%-20% reported in previous literature.^27-29^ CD30 expression may present a potential target for therapeutic intervention.

FL was the second most prevalent NHL in our study, comprising 7.9% of all patients with lymphoma, lower than the 10%-20% reported in Western countries.^27,30-34^ Consistent with a previous study,^34^ most of the FL tumors in this study were low-grade.^34^ Among the transformed DLBCL in this study, a significant majority (90.3%) were found to have originated from FL, followed by MZL, MCL, and small lymphocytic lymphoma.

Within the category of T-cell lymphoma, we discovered the majority of patients were extranodal NK/T-cell lymphoma, nasal type, followed by ALCL, T-ALL, and peripheral T-cell lymphoma, not otherwise specified (PTCL-NOS). This distribution mirrors previous findings in Asian populations, whereas in Central and South America, extranodal NK/T-cell lymphoma, nasal type, is less prevalent.^21,35^ Extranodal NK/T-cell lymphoma, nasal type, is more common in Asia, while PTCL-NOS is more prevalent in Western countries, possibly because of genetic polymorphisms and higher rates of Epstein-Barr virus (EBV) infections in Asia.^5^ Compared to a previous Asian study that reported a 24.7% frequency of angioimmunoblastic T-cell lymphoma (AITL), this research found a lower frequency of AITL (6.4%). This may be due to the limited availability of T-cell markers, which resulted in misclassification as UCT, including both patients that are ALK-positive and ALK-negative, represented 23% of T-cell lymphomas, higher than the 13.1% reported in previous Asian research patients that are ALK-positive were slightly more common, consistent with previous reports indicating a better prognosis for patients that are ALK-positive.^35-37^

A male predominance was observed across most lymphoma types, with the highest male-to-female ratio seen in MCL and NK/T-cell, nasal type, aligning with previous research on Swedish populations.^37-39^ Estrogen may offer a protective effect in lymphoproliferative diseases, although the mechanism remains controversial.^40^ Age distribution patterns were also consistent with earlier studies: B-cell lymphomas were most common in the 50-59 age group and T-cell lymphomas in the 40-49 age group. In comparison with other B-cell lymphomas, MCL peak incidence occurred in the 60-69 age group. This finding is similar to a previous study from China.^41^ Burkitt lymphoma showed a unique bimodal age distribution with peaks at <10 and 30-39 years, similar to findings from multicontinental studies.^42,43^ Previous research has demonstrated that there is a correlation between the prevalence of EBV-positive Burkitt lymphoma and advancing age.^44^ This suggests the presence of a unique biologic mechanism that varies across different age groups.^45^

In our study, 53.3% of lymphomas were found in extranodal sites, higher than the previously reported 30%.^46^ This aligns with the findings by Das et al,^47^ which reported extranodal involvement in 78.3% of patients. The head and neck, especially the tonsils and upper respiratory tract, were the most affected regions, and this finding supports previous research.^47-49^ According to the WHO classification, MZL includes extranodal (EMZL), nodal (NMZL), and splenic SMZL types.^50^ Our data show the ocular region as the most common site for EMZL, with the stomach affected less frequently, similar to previous studies.^50,51^ Similar to the finding of a big single-center study in France,^51^ cervical region is the most frequently affected site of nodal involvement, followed by the inguinal and axillary nodes.

Over a 15-year period, our institution has observed an upward trend in patients with lymphoma. As a leading national referral center, we receive patients from across the country, with 52.5% being referrals from regions outside Jakarta, including Java, Sumatra, Sulawesi, Kalimantan, Bali, and Nusa Tenggara. However, as a single-center study, these findings may not fully represent broader national trends in Indonesia. A significant portion of the population is between age 15 and 35 years, a demographic that is expected to drive an increase in lymphoma incidence over the next 5 years. The increasing trend may be supported by enhanced screening programs and the growing public awareness of cancer, leading to earlier detection, and a higher number of diagnosed patients.

The incidence of cancer in Indonesia is likely shaped by several social determinants of health that are similar to those seen in other low- to middle-income countries in Southeast Asia.^52^ In rural areas, Indonesia still struggles with low health literacy and limited access to hospital care.^53,54^ This challenge is compounded by the centralization of health care services, primarily concentrated in metropolitan areas on Java Island.^55^ This study showed peak incidence in 2014, 2019, and 2021. The introduction of national health coverage in 2014, during which the majority of patients with lymphoma in our study received national health insurance, may be the cause of the increase. In 2019, the rising may be attributed to a major health campaign and health care service initiatives launched by the government. However, the number of patients declined in 2020 because of the COVID-19 pandemic but increased again in 2021 as more individuals sought medical care. The decline in 2023 might be attributed to the emergence of private laboratories offering basic lymphoma panels.

The fitted models accurately predicted incidence trends for each lymphoma type. Projections indicate that incidence rates for most lymphoma types will continue to rise through 2028. This projection aligns with the global trend of increasing cancer patients and highlights the need for comprehensive strategies to address the growing burden of lymphoma in Indonesia. In contrast to the findings of Chu et al,^1^ which demonstrated a significant increase in patients with lymphoma among women, our study did not observe a significant difference in the increase of patients with lymphoma between men and women.^11^In addition to this, the incidence of NHL has demonstrated a consistent upward trend globally over the past few decades, as substantiated by numerous studies.^2,56,57^ Studies focusing on specific regions such as Taiwan, Japan, and South Korea have similarly reported significant increases in patients with FL, with notable annual percentage changes in incidence rates.^56^ From 2008 to 2017, there was a significant rise in FL incidence in South Korea, along with lymphoma in general.^56,57^

Despite the improved detection of patients with lymphoma in Indonesia, the country still faces limitations in the availability of comprehensive ancillary diagnostic tools and tailored management strategies that better align with prognostic outcomes. Despite these improvements, Indonesia still faces significant limitations in diagnostic tools and management strategies. Rituximab plus cyclophosphamide, doxorubicin, vincristine, and prednisone remains the most effective treatment but is limited by cost and accessibility.^58-60^ Brentuximab vedotin, effective for relapsed and refractory HL and NHL, is also limited by cost and lack of national health insurance coverage.^58^ Brentuximab vedotin, which acts as a CD30 inhibitor, has emerged as the effective therapy for relapsed and refractory patients in both HL and NHL.^61^ Despite being one of the two hospitals in Indonesia with an enhanced IHC panel, our institution has performed CD30 IHC examination on only 23.8% (1,095/4,592) of patients with DLBCL. Future studies regarding the different treatment strategies in lymphoma is still needed.

Although this study provides crucial insights into the epidemiology of lymphoma in Indonesia, limitations include the unavailability of comprehensive clinical data, such as lymphoma stage and treatment details, and limited access to IHC markers, which constrains diagnostic accuracy. These findings highlight the need for tailored guidelines that consider resource constraints.

In conclusion, our research underscores the growing burden of lymphoma in Indonesia and the need for enhanced diagnostic capabilities and strategies to manage this disease effectively. Further studies are essential to improve understanding and address the increasing incidence of lymphoma in Indonesia.

## Data Availability

The data sets generated during and/or analyzed during the current study are available from the corresponding author upon reasonable request.
